# Clinical Impact of Single and Dual Antiplatelet Therapy Beyond 12 Months on Ischemic Risk in Patients With Acute Myocardial Infarction

**DOI:** 10.3389/fcvm.2021.783344

**Published:** 2021-11-24

**Authors:** Ji Woong Roh, SungA Bae, Yongcheol Kim, Nak-Hoon Son, Deok-Kyu Cho, Jung-Sun Kim, Byeong-Keuk Kim, Donghoon Choi, Myeong-Ki Hong, Myung Ho Jeong, Yangsoo Jang, the KAMIR-NIH Investigators

**Affiliations:** ^1^Division of Cardiology, Department of Internal Medicine, Yongin Severance Hospital and Cardiovascular Center, Yonsei University College of Medicine, Yongin, South Korea; ^2^Division of Biostatistics, Yongin Severance Hospital, Yonsei University College of Medicine, Yongin, South Korea; ^3^Division of Cardiology, Severance Cardiovascular Hospital, Yonsei University College of Medicine, Seoul, South Korea; ^4^Division of Cardiology, Department of Internal Medicine, Chonnam National University Hospital, Chonnam University College of Medicine, Gwangju, South Korea; ^5^Department of Cardiology, CHA Bundang Medical Center, CHA University School of Medicine, Seongnam, South Korea

**Keywords:** acute myocardial infarction, antiplatelet therapy, aspirin, clopidogrel, major adverse cardiac and cerebral event

## Abstract

**Background:** There is ongoing debate regarding the optimal antiplatelet strategy beyond 12 months in patients with acute myocardial infarction (AMI) who undergo successful percutaneous coronary intervention (PCI). This study therefore aimed to investigate the clinical outcomes of single (SAPT) vs. dual antiplatelet therapy (DAPT) beyond 12 months in patients with stable AMI and second-generation drug-eluting stent (DES) implantation.

**Methods:** Of 13,104 patients from the Korea Acute Myocardial Infarction Registry-National Institutes of Health database, we selected 4,604 patients who underwent PCI with second-generation DES and exhibited no adverse clinical events within 12 months; they were classified into SAPT (aspirin or clopidogrel) or DAPT (aspirin and clopidogrel) groups. The primary endpoints were major adverse cardiac and cerebrovascular events (MACCE), including the composite of all-cause death, myocardial infarction (MI), and stroke between 12 and 36 months.

**Results:** The SAPT group (*n* = 1,862) was associated with a significantly lower risk of MACCE between 12 and 36 months [4.2 vs. 8.5%, hazard ratio (HR): 0.47, 95% confidence interval (CI): 0.37–0.61; *p* < 0.001] than the DAPT group (*n* = 2,742). The results were consistent after adjusting for confounders through multivariable and propensity score matching analysis. Moreover, in patients with complex features (defined as an unprotected left main PCI, implanted stent length of ≥38 mm, multivessel PCI, or ≥3 stents per patients), the SAPT group (*n* = 678) also demonstrated a significantly lower risk of MACCE between 12 and 36 months (4.9 vs. 9.9%, HR: 0.46, CI: 0.31–0.68, *p* < 0.001) than the DAPT group (*n* = 1,167).

**Conclusions:** In patients with AMI who underwent successful PCI with second-generation DES and exhibited no adverse clinical events within 12 months, the use of SAPT was associated with a significantly lower MACCE between 12 and 36 months compared with the use of DAPT.

## Introduction

The current guidelines recommend at least 12 months of dual antiplatelet therapy (DAPT)—including aspirin with a potent P2Y_12_ inhibitor, such as ticagrelor or prasugrel—and lifelong antiplatelet therapy for patients with acute coronary syndrome (ACS) undergoing percutaneous coronary intervention (PCI) with drug-eluting stents (DESs) (1, 2). After 12 months of DAPT treatment, the guidelines recommend determining whether to continue DAPT, or change to single antiplatelet therapy (SAPT) by considering the risk of coronary ischemic events in individual patients (3–6). However, in recent randomized trials involving the development of DESs, evidence for short-term DAPT has increased, with favorable ischemic outcomes observed in patients with ACS who undergo second-generation DES implantation (7, 8). Patients with ACS remained at an increased long-term risk of ischemic events (9, 10); however, there are no dedicated studies regarding the optimal antiplatelet strategy beyond 12 months (SAPT vs. DAPT) in the setting of acute myocardial infarction (AMI). Moreover, it is not easy to determine the optimal strategy by performing risk stratification for each patient in daily practice; therefore, we aimed to investigate the clinical outcomes of SAPT vs. DAPT beyond 12 months in patients with AMI who underwent successful second-generation DES implantation, using a dedicated AMI registry.

## Methods

### Study Protocols and Population Selection

The study population was selected from the nationwide, multicenter, prospective Korea Acute Myocardial Infarction Registry-National Institutes of Health (KAMIR-NIH) registry. Twenty tertiary university cardiovascular centers in Korea were recruited between November 2011 and December 2015 (Supplementary Table 1); the detailed study protocols have been published previously (11). Trained research coordinators at each center collected patient data using a web-based report form on the Internet-based Clinical Research and Trial management system, supported by a grant from the Korea Centers for Disease Control and Prevention since November 2011 (iCreaT study No. C110016). The study protocols were approved by the institutional review board of each participating center and complied with the principles of the Declaration of Helsinki (IRB approval number: CNUH-2011-172). Informed consent was obtained from all patients for participation in the KAMIR registry. The standardized definitions of all variables were unified and determined by the KAMIR-NIH committee board.

**Figure 1:**
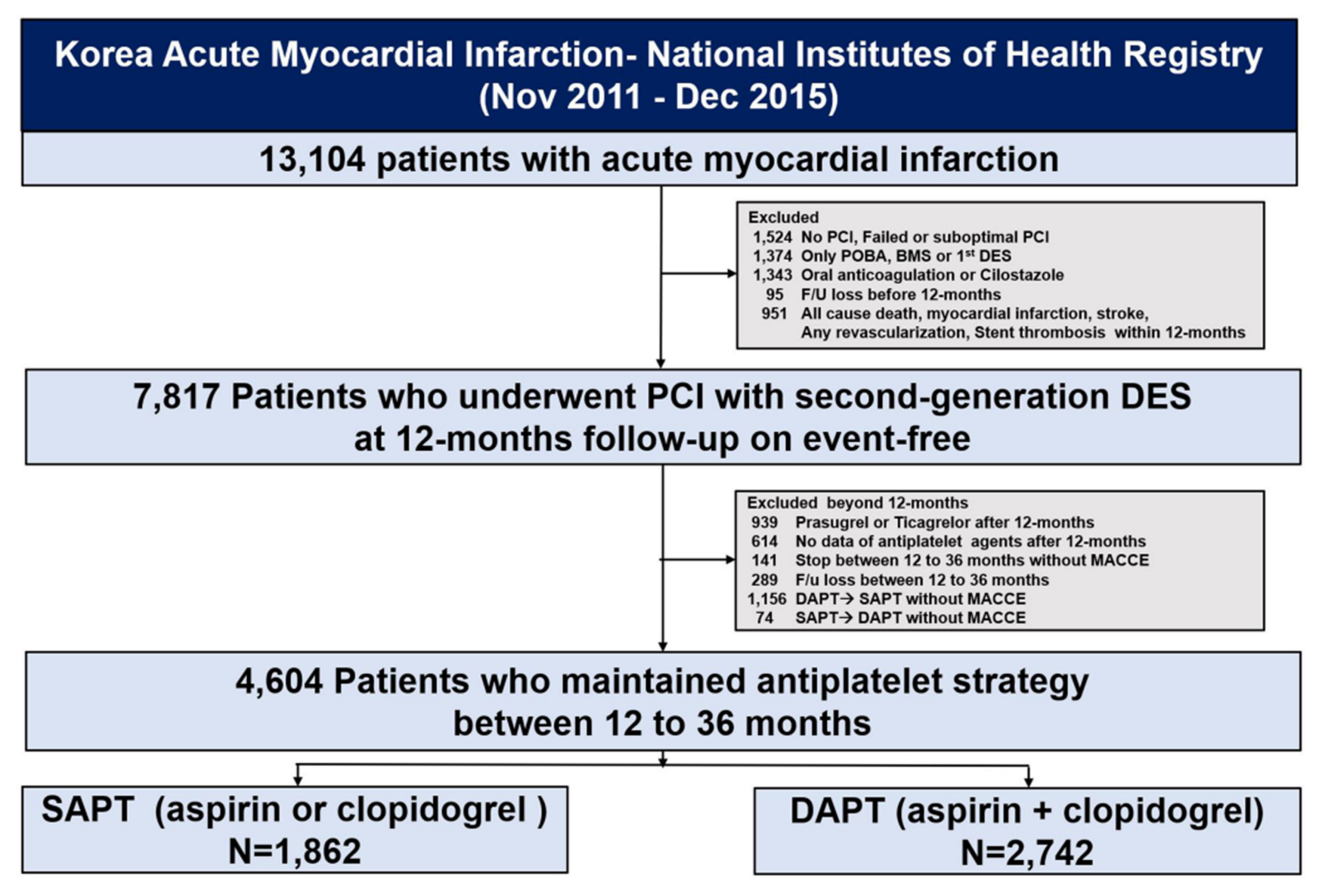
Study flowchart. PCI, percutaneous coronary intervention; POBA, plain old balloon angioplasty; BMS, bare-metal stent; DES, drug-eluting stents; PLT, platelet; MACCE, major adverse cardiac and cerebrovascular events; SAPT, single antiplatelet therapy; DAPT, dual antiplatelet therapy.

The selection process of this study population is shown in Figure 1; among 13,104 patients enrolled in the KAMIR-NIH registry, we selected patients with AMI who underwent successful second-generation DES implantation without exhibiting adverse clinical events—including all-cause death, myocardial infarction (MI), stroke, revascularization, and stent thrombosis—within 12 months. The exclusion criteria included no PCI, failed PCI, or suboptimal PCI defined as the failure to restore optimal blood flow in the infarct-related coronary artery with >50% residual stenosis and did not achieve Thrombolysis In Myocardial Infarction 2 flow after PCI; plain old balloon angioplasty alone; PCI with bare-metal stent or first-generation DES; oral anticoagulation or cilostazole use; and follow-up loss before 1 year. Additionally, we excluded patients with prolonged use of potent P2Y_12_ inhibitors, including prasugrel or ticagrelor, between 12 and 36 months; no data or terminated antiplatelet agent treatment; and who switched between DAPT and SAPT without adverse clinical events. We analyzed the clinical outcomes 12–36 months after the index procedure, stratified by antiplatelet strategy.

### Study Procedures

All patients with AMI were managed according to practical guidelines (12, 13). Patients received loading doses of antiplatelet agents, including 300 mg aspirin and a P2Y_12_ inhibitor (clopidogrel: 300–600 mg, ticagrelor: 180 mg, or prasugrel: 60 mg) before PCI. The choice of antiplatelet agent before PCI—as well as the selection of treated vessels, stent type, interventional devices (e.g., intravascular modalities including intravascular ultrasound, optical coherence tomography, fractional flow reserve, and thrombosuction), and the use of glycoprotein IIb/IIIa inhibitors—was at the operator's discretion. After PCI, daily aspirin (100 mg) and P2Y_12_ inhibitors (clopidogrel: 75 mg once, ticagrelor: 90 mg twice, or prasugrel: 10 mg once daily) were prescribed.

### Study Endpoints and Definitions

The primary endpoint was major adverse cardiac and cerebrovascular events (MACCE) between 12 and 36 months after the index procedure, defined as the composite of all-cause death, MI, and stroke. All-cause death was regarded as cardiac death unless a definite, non-cardiac cause could be identified. MI was defined according to evidence of myocardial necrosis in the vascular territory of a treated vessel, i.e., the third universal definition of MI (14). Stroke was defined as the loss of neurologic function caused by an ischemic or hemorrhagic event in the brain, with symptoms lasting at least 24 h. The secondary endpoint included individual components of MACCE and definite or probable stent thrombosis, defined according to the Academic Research Consortium definitions (15).

### Statistical Analysis

Continuous variables were compared using Student's *t*-test or Mann-Whitney *U* test, as appropriate, and are expressed as mean ± standard deviation, or median (interquartile range). Categorical variables were compared using the Chi-square or Fisher's exact test and are expressed as numbers and percentages. The cumulative incidence of each endpoint between 12 and 36 months was calculated based on Kaplan–Meier censoring estimates, whereas the comparison of clinical outcomes between the DAPT and SAPT groups was performed using the log-rank test. To correct differences in baseline and procedural characteristics, analyses were additionally performed to adjust for confounding factors. First, a multivariable Cox regression model was used for each cutoff value, with the following covariates which was significantly affect the MACCE (*p* < 0.1): age ≥ 65 years, Killip class III/IV, diabetes mellitus, glucose level, history of PCI, history of cerebrovascular accident (CVA), estimated glomerular filtration rate ≤ 60 ml/min 1.73 m^2^, left ventricular ejection fraction ≤ 50%, left main disease, and long stent ≥38 mm. Second, we performed propensity score (PS) matching between the two groups using a multiple logistic regression model. The percent standardized mean difference after PS matching was within 10% across all matched covariates, indicating a successful balance between the two groups. All statistical analyses were performed using the R statistical package version 3.6.3 software (R Foundation for Statistical Computing, Vienna, Austria) and SPSS 25.0 for Windows (IBM Corp., Armonk, NY, USA).

## Results

### Baseline Characteristics

Among the 4,604 patients with AMI who underwent PCI with second-generation DES and exhibited no adverse clinical events for 12 months, 1,862 (40.4%) and 2,742 (59.6%) patients were classified into the SAPT (aspirin, *n* = 1,347 or clopidogrel, *n* = 515) and DAPT groups, respectively. The age of the entire study population was 62.4 ± 12.1 years, and 3,562 patients (77.4%) were male; 2,317 patients (50.3%) presented with ST-elevation myocardial infarction (STEMI). The baseline clinical, lesion, and procedural characteristics of the two groups are summarized in Tables 1, 2. Patients in the SAPT group were younger, with a lower prevalence of Killip class III/IV; hypertension; diabetes mellitus; and previous history of MI, PCI, and CVA than the DAPT group. Regarding lesion and procedural characteristics, the SAPT group had a lower prevalence of multivessel disease, left main disease, ACC/AHA B2/C lesions, and glycoprotein IIb/IIIa inhibitor use; conversely, they had a higher prevalence of transradial approaches and image-guided PCI—including intravascular ultrasound or optical coherence tomography—than the DAPT group. Regarding the implanted stents, a significantly larger stent diameter, shorter total stent length, and fewer total stent numbers were observed in the SAPT group. After PS matching, the standardized differences between the groups were <10% for all variables, indicating proper matches. No significant differences existed regarding the baseline, lesion, and procedural characteristics between the groups in the PS-matched population.

**Table 1:** Baseline clinical characteristics of the study population.

	**Crude population**			**PS-matched population**			
	**SAPT** **(*n* = 1,862)**	**DAPT** **(*n* = 2,742)**	***p-*** **value**	**SAPT** **(*n* = 1,862)**	**DAPT** **(*n* = 1,862)**	***p-*** **value**	**SMD** **(%)**
**Demographics**							
Age, years	61.6 ± 11.8	63.2 ± 12.2	<0.001	61.6 ± 11.8	61.8 ± 12.1	0.143	4.87
Male	1454 (78.1%)	2108 (76.9%)	0.354	1454 (78.1%)	1437 (77.2%)	0.529	2.21
Female	408 (21.9%)	634 (23.1%)		408 (21.9%)	425 (22.8%)		
Body mass index	24.1 ± 3.1	24.2 ± 3.3	0.914	24.1 ± 3.1	24.2 ± 3.3	0.887	0.48
Killip class III/IV	138 (7.4%)	285 (10.4%)	0.001	138 (7.4%)	147 (7.9%)	0.622	1.85
STEMI	957 (51.4%)	1360 (49.6%)	0.243	957 (51.4%)	951 (51.1%)	0.870	0.64
**Cardiovascular risk factors**							
Hypertension	850 (45.6%)	1349 (49.2%)	0.020	850 (45.6%)	865 (46.9%)	0.450	2.59
Diabetes mellitus	385 (20.7%)	761 (27.8%)	<0.001	385 (20.7%)	415 (22.3%)	0.247	3.98
Glucose, mg/dL	157.3 ± 66.2	164.8 ± 73.5	<0.001	157.3 ± 66.2	159.4 ± 64.7	0.321	3.23
Dyslipidemia	241 (12.9%)	306 (11.2%)	0.074	241 (12.9%)	226 (12.1%)	0.488	2.40
Total cholesterol, mg/dL	186.3 ± 43.9	184.7 ± 43.6	0.231	186.3 ± 43.9	185.3 ± 43.4	0.138	4.85
Triglyceride, mg/dL	143.8 ± 118.6	138.1 ±127.9	0.091	143.8 ± 118.6	139.6 ± 130.2	0.262	3.91
HDL cholesterol, mg/dL	43.8 ± 11.3	43.1 ± 11.9	0.047	43.8 ± 11.3	43.5 ± 11.7	0.352	3.13
LDL cholesterol, mg/dL	117.1 ± 37.6	115.7 ± 39.2	0.199	117.1 ± 37.6	116.0 ± 36.0	0.628	1.59
Current smoker	824 (44.3%)	1116 (40.7%)	0.018	824 (44.3%)	806 (43.3%)	0.574	1.95
Family history of CVD	128 (6.9%)	200 (7.3%)	0.258	128 (6.9%)	128 (6.9%)	1.000	0.21
Previous history of MI	56 (3.0%)	180 (6.6%)	<0.001	56 (3.0%)	69 (3.7%)	0.275	4.09
Previous history of PCI	50 (2.7%)	119 (4.3%)	0.004	50 (2.7%)	61 (3.3%)	0.335	3.65
Previous history of CVA	75 (4.0%)	147 (5.4%)	0.045	75 (4.0%)	83 (4.5%)	0.569	2.19
LVEF (%)	53.1 ± 9.7	52.5 ± 10.6	0.051	53.1 ± 9.7	53.3 ± 10.8	0.708	1.35
Chronic kidney disease (eGFR, ml/min/1.73 m^2^ ≤ 60)	606 (32.5%)	956 (34.9%)	0.110	606 (32.5%)	616 (33.1%)	0.753	1.15
**Medication at discharge**							
DAPT	1853 (99.5%)	2734 (99.7%)	0.421	1853 (99.5%)	1854 (99.6%)	0.902	0.77
Aspirin	1860 (99.9%)	2740 (99.9%)	1.000	1860 (99.9%)	1860 (99.9%)	1.000	0.00
P2Y12 inhibitor	1855 (99.6%)	2736 (99.8%)	0.482	1855 (99.6%)	1856 (99.7%)	0.872	0.88
Clopidogrel	1231 (66.1%)	1903 (69.4%)		1231 (66.1%)	1240 (66.6%)		
Prasugrel	211 (11.3%)	282 (10.3%)		211 (11.3%)	205 (11.0%)		
Ticagrelor	413 (22.2%)	551 (20.1%)		413 (22.2%)	411 (22.1%)		
RAS inhibitor	1546 (83.0%)	2257 (82.3%)	0.555	1546 (83.0%)	1534 (82.4%)	0.634	1.72
Beta-blocker	1654 (88.8%)	2366 (86.3%)	0.012	1654 (88.8%)	1639 (88.0%)	0.473	2.56
Statin	1800 (96.7%)	2620 (95.6%)	0.068	1800 (96.7%)	1794 (96.3%)	0.655	1.80

*Values are mean ± SD or n (%).*

*CVA, cerebrovascular accident; CVD, cardiovascular disease; DAPT, dual antiplatelet therapy; eGFR, estimated glomerular filtration rate; HDL, high-density lipoprotein; MI, myocardial infarction; MVR, multivessel revascularization; LDL, low-density lipoprotein; LVEF, left ventricular ejection fraction; PCI, percutaneous coronary intervention; PS, propensity score; RAS, renin-angiotensin system; SAPT, single antiplatelet therapy; SMD, standard mean difference; STEMI, ST-elevation myocardial infarction*.

**Table 2:** Lesion and procedural characteristics of the study population.

	**Crude population**			**PS-matched population**			
	**SAPT** **(*n* = 1,862)**	**DAPT** **(*n* = 2,742)**	***p***	**SAPT** **(*n* = 1,862)**	**DAPT** **(*n* = 1,862)**	***p***	**SMD** **(%)**
**Lesion characteristics**							
Number of vessel disease			<0.001			0.592	1.12
One-vessel disease	998 (53.6%)	1299 (47.4%)		998 (53.6%)	1002 (53.8%)		
Multivessel disease	864 (46.4%)	1443 (52.6%)	<0.001	864 (46.4%)	879 (46.2%)	0.921	0.62
Culprit vessel			0.007			0.219	1.46
LM	39 (2.1%)	99 (3.6%)		39 (2.1%)	46 (2.5%)		
LAD	857 (46.1%)	1291 (47.1%)		857 (46.1%)	865 (46.5%)		
LCX	349 (18.7%)	450 (16.4%)		349 (18.7%)	304 (16.3%)		
RCA	617 (33.1%)	902 (32.9%)		617 (33.1%)	647 (34.7%)		
ACC/AHA B2/C lesion	1556 (83.6%)	2355 (85.9%)	0.034	1556 (83.6%)	1550 (83.2%)	0.826	0.87
**Procedural characteristics**							
Trans-radial approach	775 (41.6%)	1011 (36.9%)	0.001	775 (41.6%)	744 (40.0%)	0.317	3.38
Glycoprotein IIb/IIIa inhibitor	248 (13.3%)	437 (15.9%)	0.016	248 (13.3%)	264 (14.2%)	0.475	0.58
Thrombus aspiration	549 (29.5%)	627 (22.9%)	<0.001	549 (29.5%)	510 (27.4%)	0.167	4.59
Image guided PCI ^*^	487 (26.2%)	564 (20.6%)	<0.001	487 (26.2%)	441 (23.7%)	0.081	6.23
Stent type							
BP-BES	412 (22.1%)	606 (22.1%)	<0.001	412 (22.1%)	400 (21.5%)	0.291	3.24
DP-EES	843 (45.3%)	1502 (54.8%)		843 (45.3%)	850 (45.6%)		
DP-ZES	539 (28.9%)	552 (20.1%)		539 (28.9%)	534 (28.7%)		
Other (Sirolimus, Novolimus)	68 (3.7%)	82 (3.0%)		68 (3.7%)	78 (4.2%)		
Stent diameter	3.2 ± 0.5	3.1 ± 0.4	<0.001	3.2 ± 0.5	3.1 ± 0.9	0.226	3.87
Total stent length	28.9 ± 13.3	30.0 ± 14.5	0.010	28.9 ± 13.3	29.1 ± 13.4	0.648	1.51
Total stent number	1.4 ± 0.7	1.5 ± 0.8	<0.001	1.4 ± 0.7	1.4 ± 0.8	0.331	3.87

*Values are mean ± SD or n (%).*

*ACC/AHA, American College of Cardiology/American Heart Association; BP-BES, Biodegradable polymer-biolimus eluting stents; DES, drug-eluting stent; DP-EES, Durable polymer-everolimus eluting stents; DP-ZES, Durable polymer-zotarolimus eluting stents; LAD, left anterior descending artery; LCX, left circumflex artery; LM, left main; RCA, right coronary artery.*

*Other abbreviations as in Table 1.*

* *Imaged guided PCI including intravascular ultrasound and optical coherence tomography*.

### Benefits of SAPT Than DAPT Beyond 12 Months for the Clinical Outcomes in Patients With AMI

**Figure 2:**
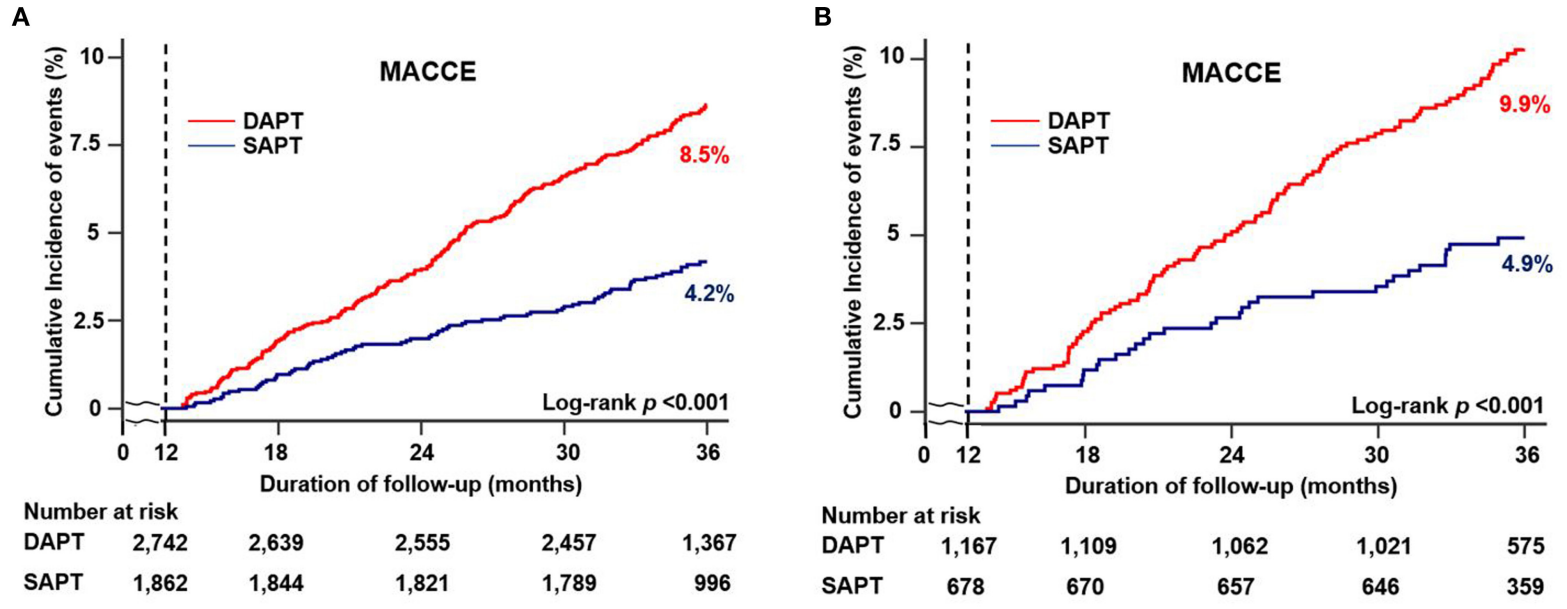
Cumulative incidence of clinical outcomes according to antiplatelet agents between 12 and 36 months. Kaplan–Meier curves comparing the rates of MACCE between SAPT and DAPT in **(A)** all patients and **(B)** patients with complex features. MACCE, major adverse cardiac and cerebrovascular events; SAPT, single antiplatelet therapy; DAPT, dual antiplatelet therapy.

**Figure 3:**
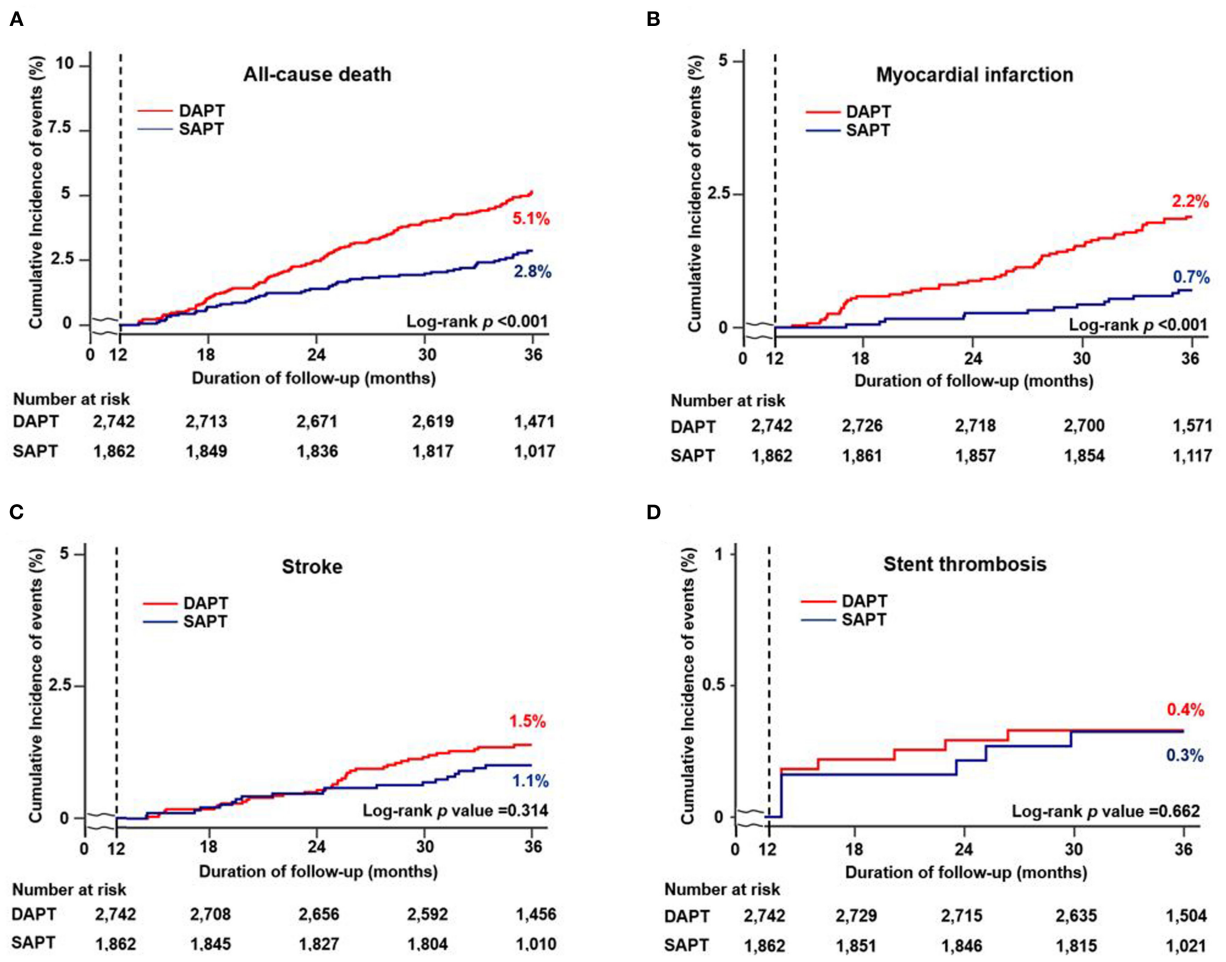
Cumulative incidence of individual clinical outcomes according to antiplatelet agents between 12 and 36 months. Kaplan–Meier curves comparing the rates of **(A)** all-cause death, **(B)** myocardial infarction, **(C)** stroke, and **(D)** stent thrombosis between SAPT and DAPT. MACCE, major adverse cardiac and cerebrovascular events; SAPT, single antiplatelet therapy; DAPT, dual antiplatelet therapy.

Figures 2, 3, and Table 3 compare the clinical outcomes between the groups; the follow-up duration was 1,102 (interquartile range: 1,060–1,140) days. The risk of MACCE between 12 and 36 months was significantly lower in the SAPT than in DAPT group [4.2 vs. 8.5%, hazard ratio (HR): 0.47, 95% confidence interval (CI): 0.37–0.61; *p* < 0.001; Figure 2A], primarily due to a significantly lower risk of all-cause death (2.8 vs. 5.1%, HR: 0.54, 95% CI: 0.40–0.74; *p* < 0.001) and MI (0.7 vs. 2.2%, HR: 0.31, 95% CI: 0.17–0.56; *p* < 0.001) in the SAPT group (Figures 3A,B; Table 3). Adjusted analysis using multivariable Cox regression and PS matching consistently demonstrated a significantly lower risk of MACCE, all-cause death, and MI in the SAPT group than in the DAPT group. There were no significant differences regarding the risk of noncardiac death, stroke, and stent thrombosis between the groups (Figures 3C,D; Table 3).

**Table 3:** Comparison of clinical outcomes.

	**SAPT** **(*n* = 1,862)**	**DAPT** **(*n* = 2,742)**	**Unadjusted**		**Adjusted^†^**		**PS-adjusted**	
			**HR (95% CI)**	***p-*** **value**	**HR (95% CI)**	***p-*** **value**	**HR (95% CI)**	***p-*** **value**
MACCE^*^	79 (4.2%)	233 (8.5%)	0.47 (0.37–0.61)	<0.001	0.54 (0.42–0.70)	<0.001	0.54 (0.41–0.71)	<0.001
All-cause death	53 (2.8%)	141 (5.1%)	0.54 (0.40–0.74)	<0.001	0.64 (0.47–0.89)	0.007	0.64 (0.45–0.90)	0.010
Cardiac death	27 (1.5%)	85 (3.1%)	0.46 (0.30–0.72)	0.001	0.57 (0.37–0.88)	0.011	0.55 (0.34–0.88)	0.012
Noncardiac death	26 (1.3%)	56 (2.0%)	0.66 (0.42–1.06)	0.084	0.75 (0.47–1.21)	0.237	0.78 (0.47–1.30)	0.340
MI	13 (0.7%)	61 (2.2%)	0.31 (0.17–0.56)	<0.001	0.33 (0.18–0.60)	<0.001	0.37 (0.19–0.69)	0.002
Target vessel MI	3 (0.2%)	20 (0.7%)	0.22 (0.06–0.73)	0.013	0.22 (0.06–0.73)	0.014	0.30 (0.08–1.07)	0.064
Non-target vessel MI	10 (0.5%)	41 (1.5%)	0.35 (0.18–0.70)	0.003	0.37 (0.18–0.80)	0.011	0.39 (0.19–0.82)	0.013
Stroke	21 (1.1%)	40 (1.5%)	0.76 (0.45–1.29)	0.315	0.83 (0.49–1.41)	0.493	0.77 (0.44–1.36)	0.369
Definite/probable ST	6 (0.3%)	11 (0.4%)	0.80 (0.30–2.17)	0.663	0.87 (0.32–2.37)	0.777	0.86 (0.29–2.55)	0.779

* *MACCE: a composite of all-cause death, MI, and stroke.*

† *Adjusted variable: age ≥ 65 years, Killip class 3/4, DM, glucose level, history of PCI, CVA, eGFR ≤ 60 ml/min/1.73 m^2^, LVEF < 50%, left main disease, long stent ≥ 38 mm.*

*Values are n (%) unless otherwise indicated.*

*HR, hazard ratio; CI, confidence interval; PS, propensity score; MACCE, major adverse cardiovascular and cerebrovascular events; ST, stent thrombosis.*

*Other abbreviations as in Table 1*.

### Clinical Benefits of SAPT Than DAPT Beyond 12 Months in Patients With AMI and Complex Features

**Figure 4:**
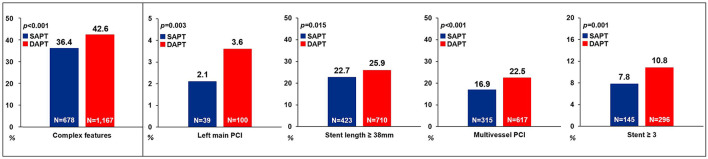
Comparison of complex features between the SAPT and DAPT groups. SAPT, single antiplatelet therapy; DAPT, dual antiplatelet therapy; PCI, percutaneous coronary intervention.

We compared the clinical outcomes by antiplatelet strategy between 12 and 36 months in patients with AMI and complex features. Considering the clinical condition of AMI and referring to the reference, the following were considered complex features: (1) unprotected left main PCI, (2) long lesion (implanted stent length ≥ 38 mm), (3) multivessel PCI, or (4) multiple stent implantation (≥3 stents per patient) (16). Of the 1,862 patients with SAPT, 678 (36.4%) had one or more complex feature, whereas among the 2,742 patients with DAPT, 1,167 (42.6%) had complex features. The SAPT group demonstrated a lower proportion of left main PCI, stent length of ≥38 mm, multivessel PCI and, ≥3 stent implantations compared with the DAPT group (Figure 4). Regarding the clinical outcomes, the SAPT group demonstrated a consistently lower rate of MACCE (4.9 vs. 9.9%, HR: 0.46, 95% CI: 0.31–0.68; *p* < 0.001) and MI (0.6 vs. 2.4%, HR: 0.24, 95% CI: 0.08–0.68; *p* < 0.007) between 12 and 36 months than the DAPT group in the adjusted analysis (Figure 2B; Table 4; Supplementary Figure 1B). Adjusted analysis showed that the risk of all-cause mortality, stroke, and stent thrombosis was not different between the two groups (Supplementary Figure 1; Table 4).

**Table 4:** Comparison of clinical outcomes in patients with complex features.

	**SAPT** **(*n* = 678)**	**DAPT** **(*n* = 1,167)**	**Unadjusted**		**Adjusted^†^**	
			**HR (95% CI)**	***p***	**HR (95% CI)**	***p***
MACCE^*^	33 (4.9%)	116 (9.9%)	0.46 (0.31–0.68)	<0.001	0.53 (0.36–0.79)	0.002
All-cause death	23 (3.4%)	73 (6.3%)	0.53 (0.33–0.85)	0.008	0.66 (0.41–1.07)	0.088
Cardiac death	11 (1.6%)	45 (3.9%)	0.42 (0.22–0.80)	0.009	0.53 (0.27–1.04)	0.066
Noncardiac death	12 (1.8%)	28 (2.4%)	0.72 (0.37–1.41)	0.337	0.76 (0.42–1.50)	0.256
MI	4 (0.6%)	28 (2.4%)	0.24 (0.08–0.68)	0.007	0.30 (0.10–0.85)	0.024
Target vessel MI	1 (0.1%)	11 (0.9%)	0.15 (0.02–1.18)	0.072	0.19 (0.05–1.30)	0.124
Non-target vessel MI	3 (0.4%)	17 (1.5%)	0.30 (0.09–1.01)	0.051	0.47 (0.15–1.40)	0.174
Stroke	11(1.6%)	19 (1.6%)	0.98 (0.47–2.06)	0.962	1.03 (0.48–2.18)	0.945
Definite/probable ST	2 (0.3%)	6 (0.5%)	0.57 (0.12–2.84)	0.495	0.47 (0.09–2.44)	0.370

* *MACCE: a composite of all-cause death, any MI, any stroke.*

† *Adjusted variable: age ≥ 65 years, Killip class 3/4, DM, glucose level, history of PCI, CVA, eGFR ≤ 60 mL/min/1.73 m^2^, LVEF < 50%, left main disease, long stent ≥ 38 mm.*

*Values are n (%) unless otherwise indicated.*

*Abbreviations as in Tables 1, 3*.

### Subgroup Analysis

**Figure 5:**
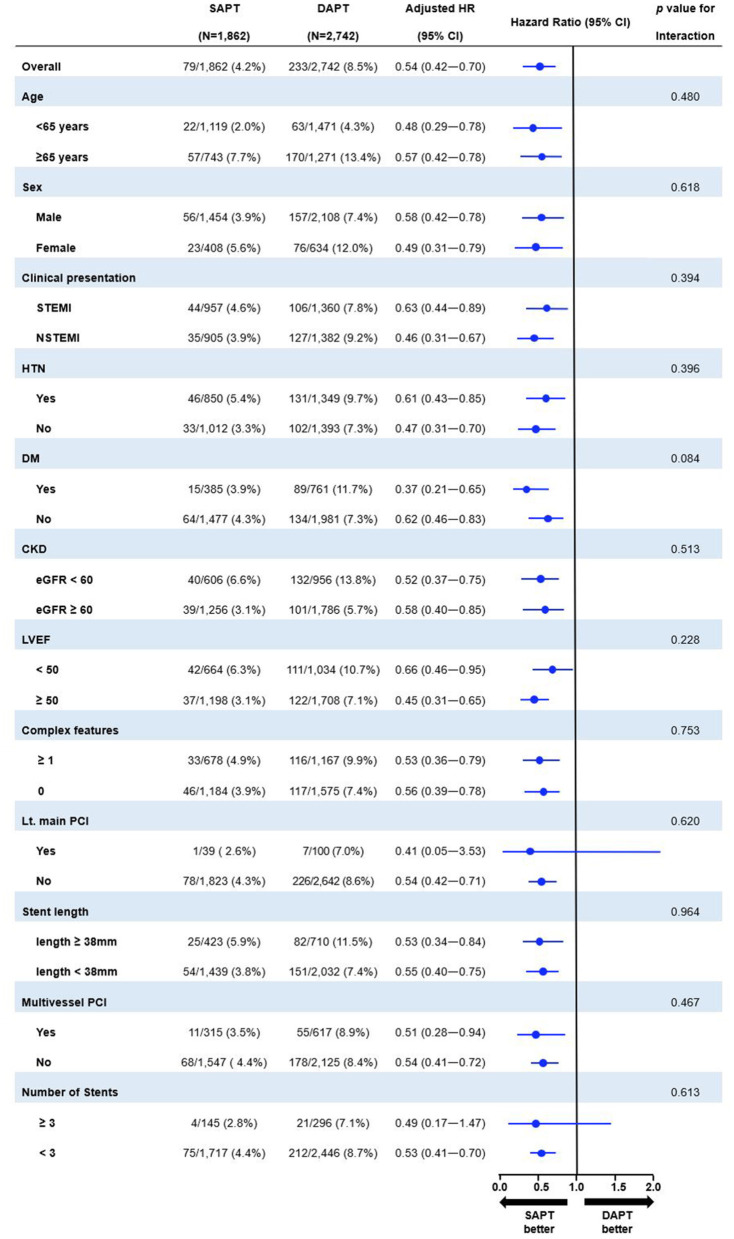
Exploratory subgroup analysis of MACCE between 12 and 36 months according to antiplatelet strategy. SAPT, single antiplatelet therapy; DAPT, dual antiplatelet therapy; HR, hazard ratio; CI, confidence interval; STEMI, ST-elevation myocardial infarction; DM, diabetes mellitus; CKD, chronic kidney disease; eGFR, estimated glomerular filtration rate; LVEF, left ventricular ejection fraction; PCI, percutaneous coronary intervention.

Figure 5 shows the prognostic impact of antiplatelet strategy beyond 12 months on the MACCE among the various subgroups using multivariable-adjusted hazard ratios. The lower risk of MACCE observed in the SAPT vs. DAPT group was consistent across all subgroups; moreover, there was no significant interaction among the subgroups.

## Discussion

**Figure 6:**
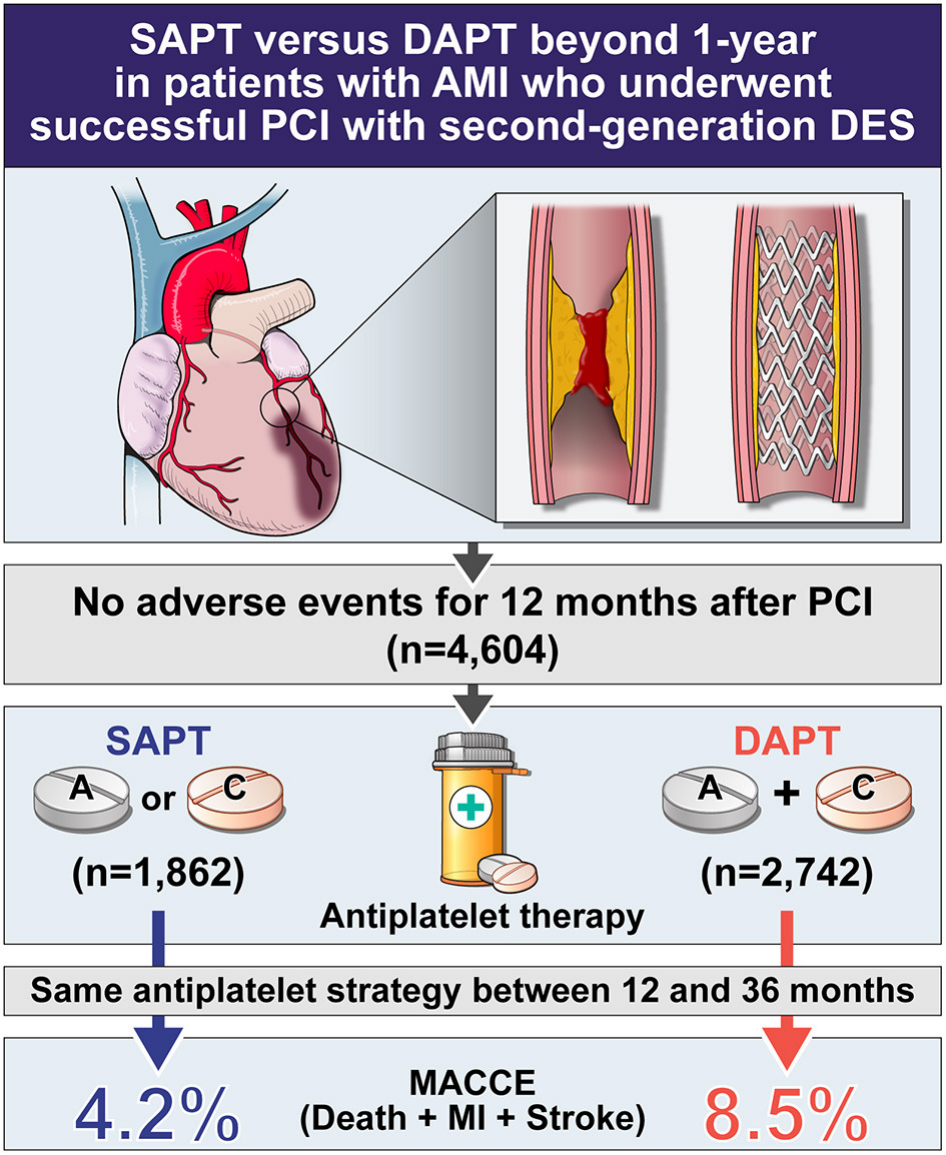
Central illustration of this study. SAPT, single antiplatelet therapy; DAPT, dual antiplatelet therapy; AMI, acute myocardial infarction; PCI, percutaneous coronary intervention; DES, drug eluting stent; MACCE, major adverse cardiac and cerebrovascular events.

This study compared clinical outcomes between 12 and 36 months according to antiplatelet strategy in patients with AMI who underwent successful second-generation DES implantation—with no clinical events within 12 months—using data from a nationwide, multicenter, dedicated AMI registry. The main findings of the study were as follows: (1) the prevalence of MACCE beyond 12 months was significantly lower in the SAPT group (Figure 6), consistently observed with confounder adjustment by multivariable analysis and PS matching; (2) in AMI with complex features, SAPT was also associated with a significantly lower risk of MACCE between 12 and 36 months than DAPT; and (3) the benefits of SAPT for MACCE were consistent across diverse subgroups, including each complex feature.

Previous randomized clinical trials—including ~20% of patients treated with PCI—did not show significant benefits with prolonged DAPT (aspirin and clopidogrel) in reducing MACCE for 28 months and possibility of bleeding compared with SAPT (aspirin) (17). However, subgroup analysis in patients with prior MI, stroke, or symptomatic peripheral artery disease demonstrated that prolonged DAPT was more effective than SAPT at reducing ischemic events (18). In another randomized trial, all patients with first- or second-generation DES implantation and prolonged DAPT using clopidogrel or prasugrel plus aspirin exhibited a significantly lower risk of stent thrombosis and MACCE than SAPT (aspirin); however, moderate-to-severe bleeding was significantly more frequent, and the rate of all-cause death was higher in the DAPT group (19). Through these two, important, large-scale randomized clinical trials, it was ascertained that prolonged DAPT did not improve mortality, while increasing bleeding events. Furthermore, a meta-analysis of six trials including 21,457 patients with DES implantation demonstrated no benefit of prolonged DAPT in patients with stable coronary artery disease regarding overall ischemic outcomes, even MI (20).

In this study, the prolonged DAPT (aspirin and clopidogrel) group did not exhibit benefits regarding MACCE, death, or MI. SAPT (aspirin or clopidogrel) demonstrated favorable clinical outcomes, and with the development of DES profile, the duration of DAPT maintenance is gradually decreasing. Unlike other studies, we only targeted patients with a second-generation DES, with a thinner strut thickness, and biocompatible or biodegradable polymer coating, to improve arterial healing and decrease the risk of thrombotic adverse events (21); this may reduce the need for prolonged intense platelet inhibition beyond 12 months. Our study was additionally analyzed in an East Asian population, with a lower number of thrombotic events observed than in Westerners (22). In a previous randomized trial including Korean patients who underwent PCI (primarily first-generation DES) exhibiting no adverse clinical events within 12 months, prolonged DAPT (aspirin and clopidogrel) for 12–36 months resulted in a small number of clinical events—including all-cause death, MI, and stroke—without associated benefits (23). Fewer absolute ischemic events observed in East Asians compared with Westerners may therefore reduce the need for prolonged DAPT. We analyzed only aspirin or clopidogrel, which are commonly used in real-world clinical practice. Since the use of DAPT including potent P2Y_12_ inhibitors (including ticagrelor and prasugrel) beyond 12 months was excluded, the effect of reducing the ischemic risk of DAPT may be less prominent than in other studies; therefore, in the second-generation era, a large-scale, randomized study to determine the optimal antiplatelet strategy in patients—including Asians and Westerners with stable AMI beyond 12 months—is needed.

Additionally, the SAPT group exhibited favorable clinical outcomes in patients with complex features. Along with improvements in DES profile and adjunct pharmacological treatments, patients with complex lesions could now undergo treatment via PCI in more cases. Although there remains no clear definition of complex features for PCI, several studies and expert consensus commonly include bifurcation with two stents, chronic total occlusion, severely calcified lesions using rotational atherectomy, unprotected left main PCI, implanted long stent, multivessel PCI, and ≥3 stents implanted (4, 24, 25). Nevertheless, there are limited data regarding the appropriate antiplatelet strategy for complex PCI.

Recently, short DAPT with ticagrelor monotherapy exhibited significantly lower bleeding events, without increasing the ischemic risk in patients with complex PCI or STEMI (24, 26); however, in real-world AMI settings, there is still no concept of complex PCI. Furthermore, chronic total occlusion, or bifurcation with two stents or rotational atherectomy, is rare; therefore, we only included left main PCI, implanted long stent, multivessel PCI, and ≥3 implanted stents, as components of complex features in AMI. Nevertheless, prolonged DAPT did not reduce ischemic risk compared with SAPT. Considering the reduction in the risk of ischemic events, our study results suggest that SAPT could be considered for patients with AMI and complex features who underwent second-generation DES implantation without adverse clinical events for 12 months.

This study has some limitations; first, there is an inherent limitation regarding nonrandomized, observational, registry data, which might have resulted in selection bias. However, we attempted to adjust for the measured confounders of different baseline characteristics through the adjusted analyses, including PS matching. Second, covariates used in PS matching were mainly derived from data during the index hospitalization, which did not allow adjustments for the differences in patient characteristics between 12 and 36 months after index hospitalization. Third, since there is no record of bleeding events beyond 12 months, it was difficult to analyze the extent to which bleeding events affect MACCE, including all-cause death. Our study therefore focused on ischemic risk reduction according to antiplatelet strategy beyond 12 months; complex features with a high ischemic burden were analyzed separately. Moreover, among P2Y_12_ inhibitors, only clopidogrel was selected for this analysis beyond 12 months due to less bleeding tendency and most used in real world practice. Fourth, the definite reasons for DAPT vs. SAPT selection beyond 12 months is unclear, as it was decided by each clinician considering the balance of ischemic and bleeding risk in individual patients. Fifth, this study was conducted in a dedicated east Asian population and the result of this current study cannot be extrapolated to other ethnicities. Lastly, the KAMIR-NIH registry was designed for 3 years of follow-up; thus, we did not evaluate long-term ischemic risks beyond 3 years.

In conclusion, compared with DAPT with clopidogrel, SAPT using aspirin or clopidogrel alone demonstrated a significantly lower risk of MACCE between 12 and 36 months in patients with AMI treated with second-generation DES who remained uneventful for 12 months. Moreover, a reduction in ischemic risk was also observed for AMI with complex features in the SAPT group. Large-scale, multinational randomized trials are thus required to identify the optimal antiplatelet strategy beyond 12 months in patients with AMI in the second-generation DES era.

## Data Availability Statement

The original contributions presented in the study are included in the article/Supplementary Material, further inquiries can be directed to the corresponding author.

## Ethics Statement

The studies involving human participants were reviewed and approved by Chonnam National University Hospital Institutional Review Board, IRB approval number: CNUH-2011-172. The patients/participants provided their written informed consent to participate in this study.

## Author Contributions

JR and YK: study concept and design, interpretation of results, and preparation of manuscripts. JR, SB, YK, and MJ: data collection. JR, SB, YK, and N-HS: data analysis and statistics. D-KC, J-SK, B-KK, DC, M-KH, MJ, and YJ: validation. YK, D-KC, J-SK, B-KK, D-KC, M-KH, MJ, and YJ: supervision. JR, SB, YK, D-KC, J-SK, B-KK, DC, M-KH, MJ, and YJ: manuscript review and editing. All authors contributed to the article and approved the submitted version.

## Funding

This study was supported by a faculty research grant of Yonsei University College of Medicine (6-2020-0161) and research seed money of Internal Medicine in Yongin Severance Hospital.

## Conflict of Interest

The authors declare that the research was conducted in the absence of any commercial or financial relationships that could be construed as a potential conflict of interest.

## Publisher's Note

All claims expressed in this article are solely those of the authors and do not necessarily represent those of their affiliated organizations, or those of the publisher, the editors and the reviewers. Any product that may be evaluated in this article, or claim that may be made by its manufacturer, is not guaranteed or endorsed by the publisher.
