# Supplementary material for: Clinical Impact of Single and Dual Antiplatelet Therapy Beyond 12 Months on Ischemic Risk in Patients With Acute Myocardial Infarction
Source: Front Cardiovasc Med. 2021 Nov 24;8:783344. doi: 10.3389/fcvm.2021.783344 (PMC8652119; doi:10.3389/fcvm.2021.783344)
Supplement: Supplementary file 1 [file Data_Sheet_1.docx]

**Supplementary materials**

**Clinical impact of single and dual antiplatelet therapy beyond 12 months on ischemic risk in patients with acute myocardial infarction**

**Supplement to: JW Roh, SA Bae, Y Kim et al., on behalf of the KAMIR-NIH Investigators**

**CONTENTS**

**Supplementary Figure 1. Cumulative incidence of individual clinical outcomes according to antiplatelet agents between 12 and 36 months in complex feature**

**Supplementary Table 1. KAMIR-NIH Experimental groups and Investigators**

**Supplementary Figure 1. Cumulative incidence of individual clinical outcomes according to antiplatelet agents between 12 and 36 months in complex feature**

Kaplan–Meier curves comparing the rates of (A) all-cause death, (B) myocardial infarction, (C) stroke, and (D) stent thrombosis between SAPT and DAPT

MACCE, major adverse cardiac and cerebrovascular events; SAPT, single antiplatelet therapy; DAPT, dual antiplatelet therapy


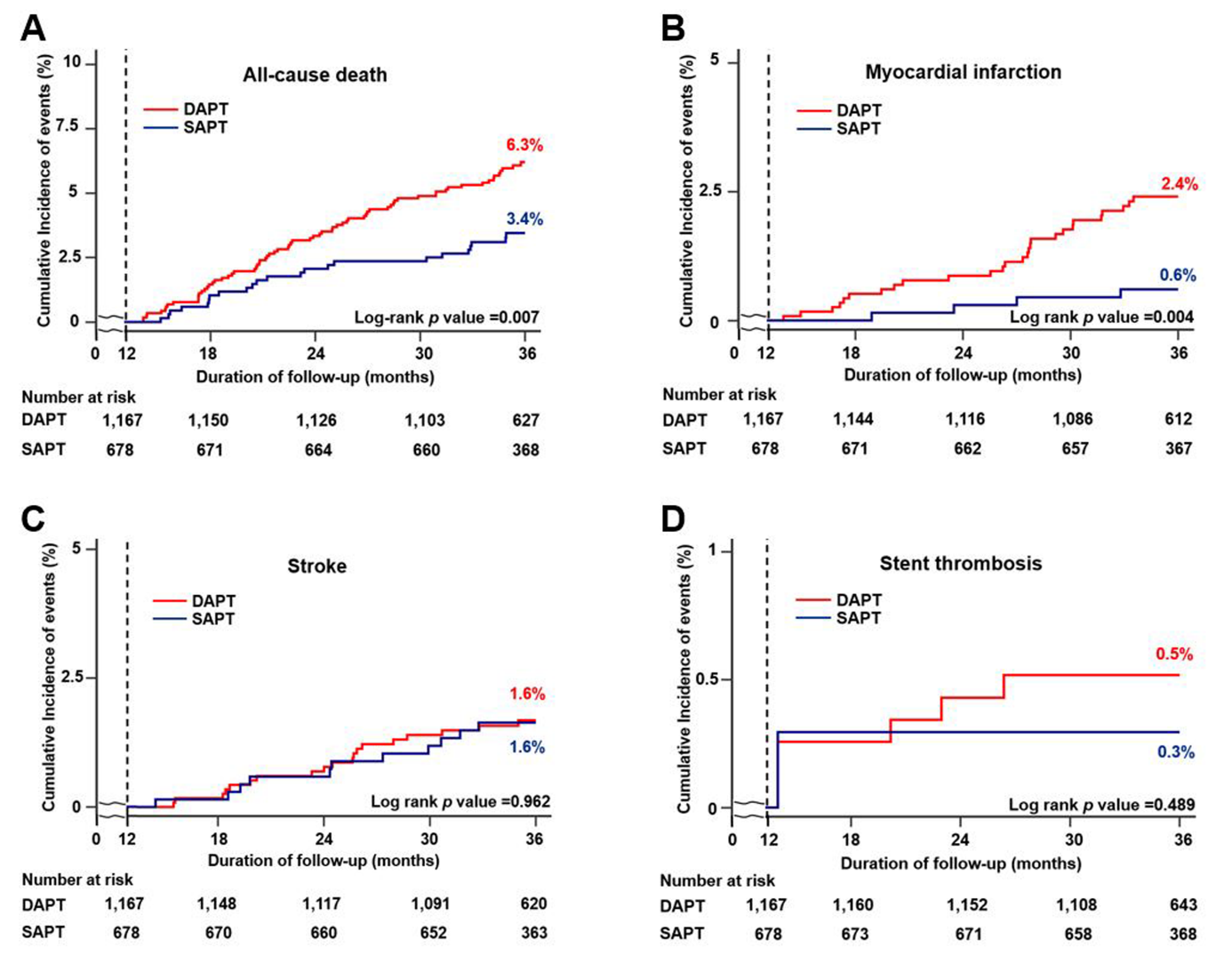


**Supplementary Table 1. KAMIR-NIH Experimental groups and Investigators**

1. Chonnam National University Hospital, Gwangju, Korea; Myung Ho Jeong, principal investigator

2. Seoul National University Hospital, Seoul, Korea; Hyo Soo Kim

3. Sungkyunkwan University, Seoul Samsung Medical Center, Seoul, Korea; Hyeon Cheol Gwon

4. The Catholic University of Korea, Seoul St. Mary’s Hospital, Seoul, Korea; Ki Bae Seung

5. Korea University Guro Hospital, Seoul, Korea; Cheol Ung Choi

6. Kyungpook National University Hospital , Daegu, Korea; Shung Chull Chae

7. Kyung Hee University Hospital at Gangdong, Seoul, Korea; Chong Jin Kim

8. Pusan National University Hospital, Busan, Korea; Kwang Soo Cha

9. Yeungnam University Hospital, Daegu, Korea; Jong Seon Park

10. Wonju Severance Christian Hospital, Wonju, Korea; Jung Han Yoon

11. Chonbuk National University Hospital, Jeonju, Korea; Jei Keon Chae

12. Jeju National University Hospital, Jeju, Korea; Seung Jae Joo

13. Seoul National University Bundang Hospital, Seongnam, Korea; Chang Hwan Yoon

14. Keimyung University Dongsan Medical Center, Daegu, Korea; Seung Ho Hur

15. Chungnam National University Hospital, Daejeon, Korea; In Whan Seong

16. Chungbuk National University Hospital, Cheongju, Korea; Kyung Kook Hwang

17. Inje University Haeundae Paik Hospital, Busan, Korea; Doo Il Kim

18. Wonkwang University Hospital, Iksan, Korea; Seok Kyu Oh

19. Gachon University Gil Medical Center, Incheon, Korea; Tae Hoon Ahn

20. Gyeongsang National University Hospital, Jinju, Korea; Jin Yong Hwang
